# Dietary diversity among reproductive aged women attending urban and rural healthcare facilities, Middle Delta, Egypt

**DOI:** 10.1186/s12889-026-26977-2

**Published:** 2026-04-02

**Authors:** Nadira Mansour Hassan, Yasmin Abd-Elkader Elwan, Mira Maged Abu-Elenin, Eman Ali Younis

**Affiliations:** Public Health and Community Medicine Department, Tanta University, Faculty of Medicine, Tanta City, Egypt

**Keywords:** Dietary diversity, Nutritional adequacy, Reproductive-aged women, Micronutrient deficiency, Urban and rural localities, Egypt

## Abstract

**Background:**

Proper nutrition is essential for women of reproductive age (WRA) as it promotes hormonal balance, fertility, and healthy pregnancy and lactation. Dietary diversity (DD) is an important component of diet quality and Minimum dietary diversity has been widely used as a proxy indicator for micronutrient adequacy. There is a notable lack of studies in Egypt addressing dietary diversity among WRA. This study investigated the dietary diversity among those who attended an urban and a rural health care facilities in Egypt.

**Methods:**

A cross-sectional study was carried out between January 2024 and June 2025 in two healthcare facilities in El- Mahalla El- Kubra district: an urban comprehensive center and a rural primary health care unit. A total of 400 women aged 15–49 years were chosen by systematic random sampling. Data were collected by direct interviews, which included sociodemographic information, 24-hour dietary recall, and food frequency questionnaire. The statistical analysis used chi-square tests, with p-values < 5% considered significant.

**Results:**

More than half (52.5%) of participants reported inadequate DD. Urban women had considerably more DD (52.1%) than rural ones (42.2%). Lactating women had the greatest dietary diversity among urban women (60.9%), while non-pregnant, non-lactating women reported the lowest percentage of diversed diet among urban and rural participants. Women with easy access to food reported higher sufficiency (56.5%) compared to those affected by high food prices (35.3%) (*p* < 0.001). Urban women consumed more meat, fish, nuts, and vitamin A-rich fruits and vegetables, but rural women consumed more eggs and pulses. Grains were the most often consumed dietary group (98.3%). Unemployment, poor educational level, non-pregnancy and non-lactation status, and limited food availability were all significant indicators of insufficient dietary diversity.

**Conclusion:**

The study identifies significant dietary diversity gaps, notably among rural women, non-pregnant, and non-lactating women, stressing the importance of targeted nutrition interventions and socioeconomic empowerment.

**Supplementary Information:**

The online version contains supplementary material available at 10.1186/s12889-026-26977-2.

## Introduction

Dietary diversity (DD) refers to the variety of foods consumed across and within food groups over a specific period. It is widely recognized as a key indicator of diet quality, ensuring optimal intake of essential nutrients and supporting both physical and mental development. For women of reproductive age (WRA), dietary diversity and nutritional adequacy are particularly critical, as they influence overall health, hormonal balance, fertility, and pregnancy outcomes. Adequate nutrition during this life stage supports menstrual health, fetal development, and helps prevent micronutrient deficiencies that may affect both mother and child [1, 2].

Evidence from low- and middle-income countries (LMICs) indicates that dietary diversity among WRA is often inadequate. A review of studies conducted between 2011 and 2021 reported that 42.3% to 90% of women consumed poorly diversified diets, with insufficient intake of essential micronutrients [3]. For example, a 2023 study in South Africa found that only 20.6% of WRA consumed at least five food groups daily [4], while a 2021 study in western Ethiopia reported that just 43.6% of pregnant women achieved adequate dietary diversity [5].

In Egypt, data from the National Nutrition Institute (NNI) between 2015 and 2020 showed that most adult females consumed insufficient amounts of key nutrients such as potassium, calcium, magnesium, and vitamin A, while intake of sodium, copper, and vitamin B1 exceeded recommended levels. Alarmingly, potassium, calcium, and magnesium intake fell below 50% of the recommended daily allowances [6]. These micronutrient inadequacies likely reflect monotonous diets dominated by cereals and limited intake of animal-source foods, fruits, and vegetables. Such patterns may differ between urban and rural settings due to disparities in food availability, affordability, and nutrition awareness.

Global dietary guidelines emphasize the importance of consuming a wide range of foods across all food groups. A balanced diet should include vegetables, fruits, grains, dairy, and protein sources, while limiting unhealthy fats, sugars, and salt. Nutritional requirements also vary based on age, physical activity, and physiological states such as pregnancy or lactation. Women require adequate intake of macronutrients carbohydrates, proteins, and fats as well as key micronutrients like iron, folic acid, calcium, and vitamin D to support reproductive health and general wellness [7–9].

Despite existing research on general dietary patterns in Egypt, few studies have examined dietary diversity across reproductive stages and urban–rural settings using standardized measures such as the Minimum Dietary Diversity for Women (MDD-W). This gap limits understanding of context-specific factors associated with diet quality and hinders targeted interventions. Women attending healthcare facilities represent a critical group for public health interventions, as they are already engaged with health services and may benefit from nutrition counseling. Understanding their dietary patterns can inform facility-based nutrition programs.

This study aims to address these gaps by assessing dietary diversity among pregnant, lactating, and non-pregnant, non-lactating women attending urban and rural healthcare facilities in Egypt and identifying factors associated with dietary diversity.

### Objectives

1. To assess dietary diversity among women at reproductive age attending urban and rural health care facilities at the different reproductive stages.
2. To explore the pattern of consumption of the 10 food groups among WRA at both settings.
3. To identify factors associated with inadequate dietary diversity among the studied women at both settings.

### Subject and methods

#### Study design

A facility-based cross-sectional study design was employed. The study adhered to the Strengthening the Reporting of Observational Studies in Epidemiology (STROBE) guidelines to ensure transparency, methodological rigor, and reproducibility [10]. Details of STROBE checklist implementation are provided in the Supplementary Material (File 1).

### Study setting and duration

The study was conducted in two primary health care facilities: an urban comprehensive health center in El Mahalla El-Kubra City and a rural primary health care unit in Kafer Higazy village. Both facilities are located in El Mahalla El-Kubra district, the largest district in Gharbia Governorate, in the Middle Delta region of Egypt. According to the Central Agency for Public Mobilization and Statistics (CAPMAS) 2024 data, the total population of El Mahalla El-Kubra district was 826,692, including 407,004 females.

Data collection was carried out from January 2024 to June 2025. Although the extended data collection period may introduce potential seasonal variation in dietary intake, participants were recruited continuously across different months and seasons, which may have partially distributed seasonal effects across the sample. Seasonal variation was not analytically controlled for and is therefore acknowledged as a limitation of the study.

### Target population

The target population comprised women of reproductive age (15–49 years) attending the selected primary health care facilities during the study period.

### Inclusion criteria

Women aged 15–49 years who attended the selected urban or rural primary health care facilities during the data collection period were eligible for inclusion, regardless of marital status, educational level, or socioeconomic background.

### Exclusion criteria

Women with mental illness, serious medical conditions (e.g., cancer, renal failure, or hepatic failure), chronic diseases, dietary restrictions (such as food allergies or intolerances), or those reporting tobacco use, alcohol consumption, or substance use were excluded, as these factors may influence dietary behavior. Alcohol consumption is uncommon and generally socially unacceptable among women in the study population due to prevailing cultural norms. In addition, women who reported fasting on the day preceding the interview were excluded to avoid misclassification of dietary intake. Fasting was primarily related to religious practices, which may have introduced selection bias and is therefore acknowledged as a study limitation.

### Sample size and sampling technique

The minimum required sample size was calculated using the formula N = (1.96) ^2 pq / d^2, where 1.96 represents the critical value of the z-score at a 95% confidence level. The expected prevalence (p) of adequate dietary diversity among women of reproductive age in urban and rural settings was assumed to be 50%, based on previous studies [3–5]. To yield the maximum sample size. The value of q was calculated as (1 − p), and d represented the margin of error, set at 0.05. Accordingly, the minimum calculated sample size was 384 participants. To compensate for potential non-response or missing data, the sample size was increased to 400 women.

A systematic random sampling technique was applied. Based on average monthly attendance, sampling was conducted proportionally across the two facilities. A total of 213 women were recruited from the urban health center and 187 women from the rural health unit. Every fourth eligible woman was selected after a random starting point until the required sample size at each site was achieved. Although this sample represents a small proportion of the total female population of El Mahalla El-Kubra district, it was considered adequate to assess dietary diversity among women attending primary health care facilities rather than the general population.

### Data collection tools

Data were collected using a pre-designed, interviewer-administered structured questionnaire that included the following components:

#### Sociodemographic characteristics

age, residence, marital status, occupation, educational level, family income, and food accessibility.

### Dietary assessment

Dietary diversity was assessed using a single 24-hour dietary recall and an adapted dietary diversity questionnaire based on ten food groups. Food group classification was adapted from the Food and Agriculture Organization’s Minimum Dietary Diversity for Women (MDD-W) guidelines to reflect commonly consumed foods in the Egyptian context. Adaptation was informed by national dietary patterns and expert consultation to enhance contextual relevance and maintain comparability. When a food item could potentially belong to more than one food group, classification followed FAO MDD-W guidance [11, 12].

A validated food frequency questionnaire (FFQ) was used to assess the frequency of consumption of commonly consumed foods and food groups (daily, weekly, or monthly). FFQ data were used descriptively to characterize habitual dietary patterns. Quantities of foods consumed were not assessed, and FFQ data were not incorporated into dietary diversity scoring or regression analyses; this is acknowledged as a limitation [13].

- Minimum Dietary Diversity for Women (MDD-W) is a simple yes/no indicator that determines whether a woman has consumed at least five out of ten defined food groups in the previous 24 hours. Achieving this threshold suggests a greater likelihood of consuming a micronutrient-adequate diet i.e. adequate diverse diet  [12].
- The MDD-W and food group diversity score were calculated based on ten food groups. (components of these groups were adapted to reflect the nature of commonly consumed foods in the Egyptian context):1-Grains (bread, rice, pasta/noodles or other foods made from grains White root/ tuber Potatoes, taro),2-Pulses (mature beans or peas (fresh or dried seed), lentils or bean/pea products),3-Nuts and seeds (peanut, sesame or nut/seed “butters”),4-Dairy product (milk, cheese, yoghurt or other milk products but NOT including butter, ice cream, cream),5-Organ meat, meat and poultry, fish and seafood, (Liver, kidney, heart or other organ meats Luncheon – sausage -Beef, goat, duck and pigeon -rabbit, chicken -Fresh, dried fish, salted fish, canned fish and seafood) shrimp and crab),6-Eggs, 7-Dark green leafy vegetables (spinach, dark green leafy lettuce, cabbage, watercress and mukhiya), 8- Other vitamin A-rich fruits and vegetables (red pepper, carrots, sweet potatoes apricot, mango), 9- Other vegetables (cauliflower - cucumber - green pepper - - okra - green onions – tomatoes), 10- Other fruits (grapes - guava - banana – apple- tangerine, cantaloupe- orange- lemon).
- The adapted FAO dietary diversity sheet is shown at Supplementary File (2).

#### Procedure

By using the 24-hour recall method, participants were asked to describe the foods (meals and snacks) they ate the previous day, whether at home or outside the home, starting with the first food eaten in the morning. All mentioned foods and drinks were recorded. Then the dietary diversity was determine using the minimum dietary diversity for women scores (MDD-W) based on information obtained in the 24 -hour recall for each participant.

Each participant received a score of 1 for consuming any item from a given food group and 0 if not. The total dietary diversity score was calculated by summing the scores across the ten food groups. Women who consumed foods from five or more groups were classified as having adequate dietary diversity, while those who consumed fewer than five were considered to have inadequate dietary diversity.

### Validity assessment of the study tools

#### Face and content validity

The questionnaire underwent a thorough validation process to ensure its accuracy and relevance. Face validity was 85% according to the results of pooled review of three experts at public health and nutrition who reviewed the tool to assess its clarity, relevance, and wording appropriateness. Based on their feedback, modifications were made to improve the phrasing of certain items and ensure that the instructions were clear and elicited appropriate responses. Also, Alpha Cronbach’s reliability was 0.870 which indicate good internal consistency.

##### **Pilot study**

was carried out, before starting gathering study data, to test the questionnaire’s feasibility, estimate the time required to complete the questionnaire, identify possible barriers that may arise during the study’s execution and how to manage them, it included (30 women not included in study analysis). The pilot study revealed that the time needed to fill out the questionnaire ranged from 10 to 15 min.

### Ethical consideration

The current study adhered to the research ethical rules applied in Faculty of Medicine, Tanta University, throughout the whole period of implementation. Approval of the research protocol was from the Ethical Committee of Tanta Faculty of Medicine before starting the study (Approval Code: 36264MS558/4/24). The purpose of the study was explained to participants before data collection. Informed consent was obtained from all participants before conducting the study. In the case of illiterate participant, consent was obtained from their legal guardians. Also, Confidentiality and privacy were guaranteed during the whole period of the study.

### Statistical analysis

- Sorting and analysis of data were performed by using Statistical Package for Social Sciences (SPSS) version 21, For quantitative data mean standard deviation were calculated, and significance was tested whenever needed.
- For qualitative data, Chi-square Test (χ^2^) was used for comparison between two independent groups (diverse versus non-diverse diet). Monte Carlo Test was applied when the assumptions of the chi-square test were not met.
- Binary logistic regression was used to identify factors associated with inadequate dietary diversity. Model assumptions were checked: multicollinearity was assessed using Variance Inflation Factor (VIF), with all variables < 2, indicating no significant collinearity. Model fit was evaluated using the Hosmer–Lemeshow test (*p* > 0.05), confirming good fit. Minimal missing data (< 2%) were handled by complete case analysis.

## Results

**Fig. 1:**
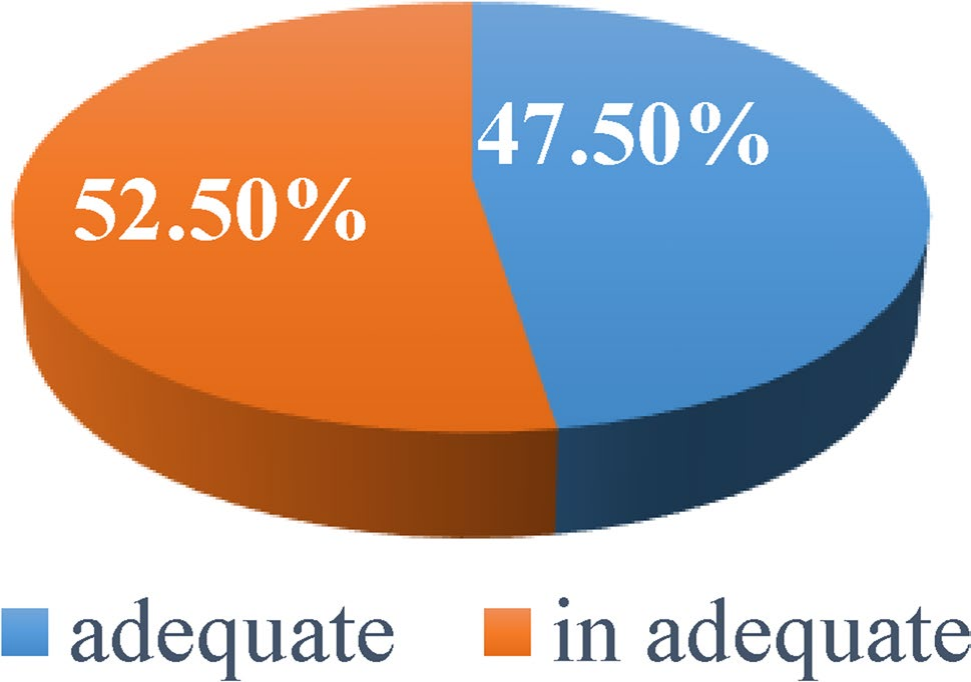
Dietary diversity among reproductive aged women attending the studied health care facilities

Total of 400 women of reproductive age were enrolled in the study, including 213 women from the urban Comprehensive Health Center and 187 women from the rural Primary Health Care Unit. Overall, 47.5% of participants achieved adequate dietary diversity, while 52.5% had inadequate dietary diversity, as shown in Fig. 1.

Table 1 presents the association between dietary diversity and sociodemographic characteristics. Adequate dietary diversity was significantly more prevalent among urban women compared with rural women (52.1% vs. 42.2%, *p* = 0.002). Higher dietary diversity was also observed among women with university education, governmental employment, sufficient household income, lactating status, and those reporting easy access to food items.

**Table 1:** Sociodemographic characteristics and dietary diversity among reproductive aged women attending urban and rural health care facilities

**Sociodemographic data**	**Adequate N=190**		**Inadequate N=210**		*x* ^2^	***p*** ** value**
	**n**	**%**	**n**	**%**		
Residence						
Urban	111	52.1	102	47.9	9.3	0.002*
Rural	79	42.2	108	57.8		
Age group					5.54	0.136
< 20	7	35.0	13	65.0		
20-	87	53.0	77	47.0		
30-	77	44.5	96	55.5		
40-	19	44.2	24	55.8		
Mean ± SD	29.89 ±6.76		30.61 ± 7.23		t (1.03)	0.30
Range	17-45		17-45			
Marital status						
Single	22	44.2	30	55.8	2.43	0.12
Married	166	50.0	170	50.0		
Ex -married	2	16.7	10	83.3		
Educational level						
Illiterate, read and write, primary	12	24.0	39	76.0		
Secondary/technical	53	38.8	85	61.2	29.8	<0.001*
University	125	60.2	86	39.8		
Occupation						
Housewife	77	41.4	114	58.6		
Manual worker	7	33.3	14	66.7	14.78	0.005*
Governmental employee	53	64.3	31	35.7		
Private employee	30	53.6	26	46.4		
Student	23	47.9	25	52.1		
Family income						
Not enough	41	34.4	79	65.6	12.5	0.002*
Just enough	123	53.5	107	46.5		
Enough and saving	26	52.0	24	48.0		
Reproductive state						
Non pregnant non lactating	108	43.5	140	56.5	10.8	0.004*
Pregnant	41	53.2	36	46.8		
Lactating	41	54.7	34	45.3		
Food stuff availability						
Available and easy to get it	130	56.5	100	43.5	15.43	<0.001*
Difficult due to high prices	60	35.3	110	64.7		

chi square test, Adequate: consume ≥ food groups /24hs Inadequate: consume < 5 food groups/24hs

*p:<0.05 (statistically significant)

**Fig. 2:**
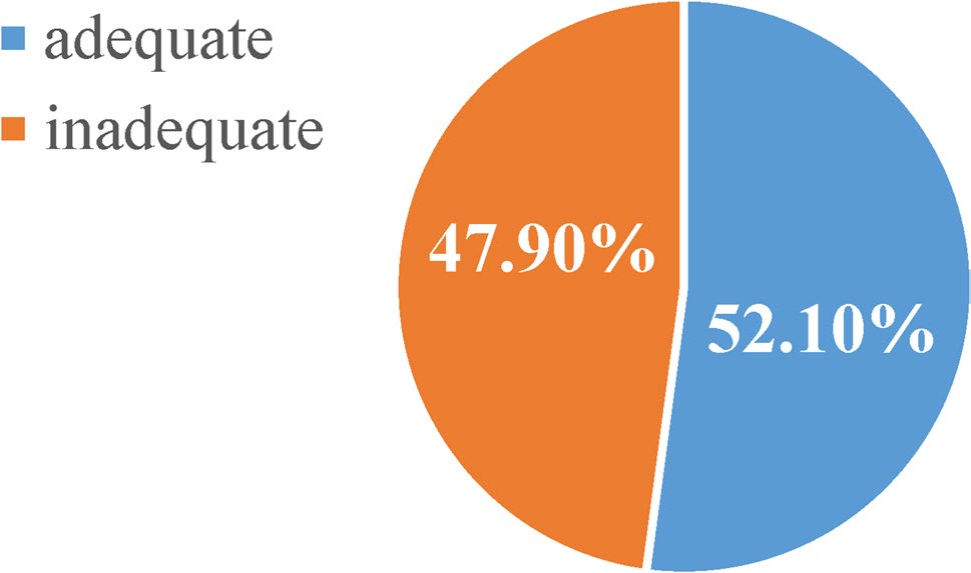
Prevalence of dietary diversity among participants attending urban health care facility

**Fig. 3:**
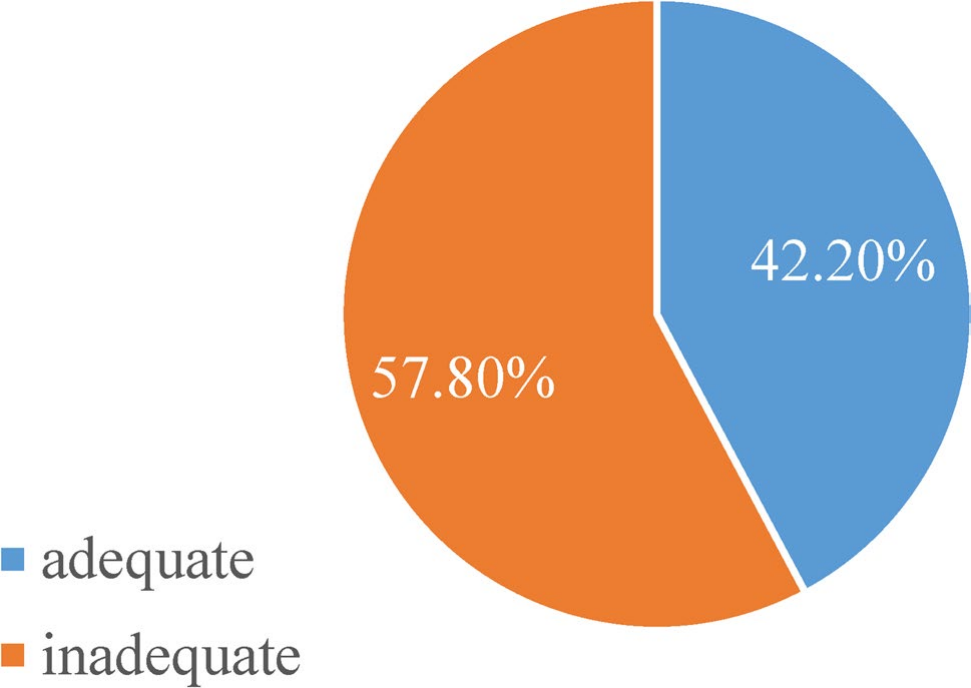
Prevalence of dietary diversity among participants attending rural health care unit.

Figures 2 and 3 show the dietary diversity among participants attending the studied urban and rural health care Facilities where urban women demonstrated significantly better dietary diversity (52.1%) compared to rural women (42.2%).

**Fig. 4:**
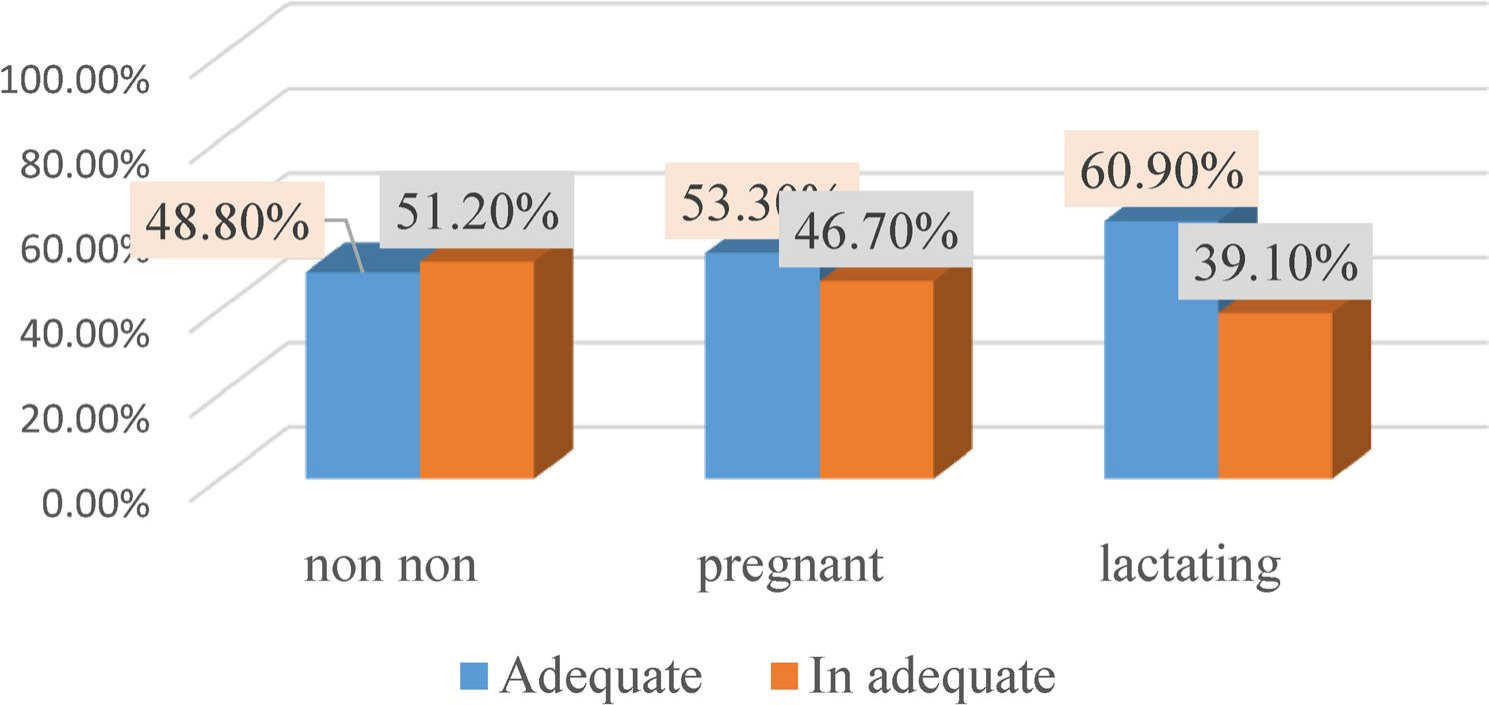
Dietary diversity among participant attending urban health care Facility according to their reproductive state

**Fig. 5:**
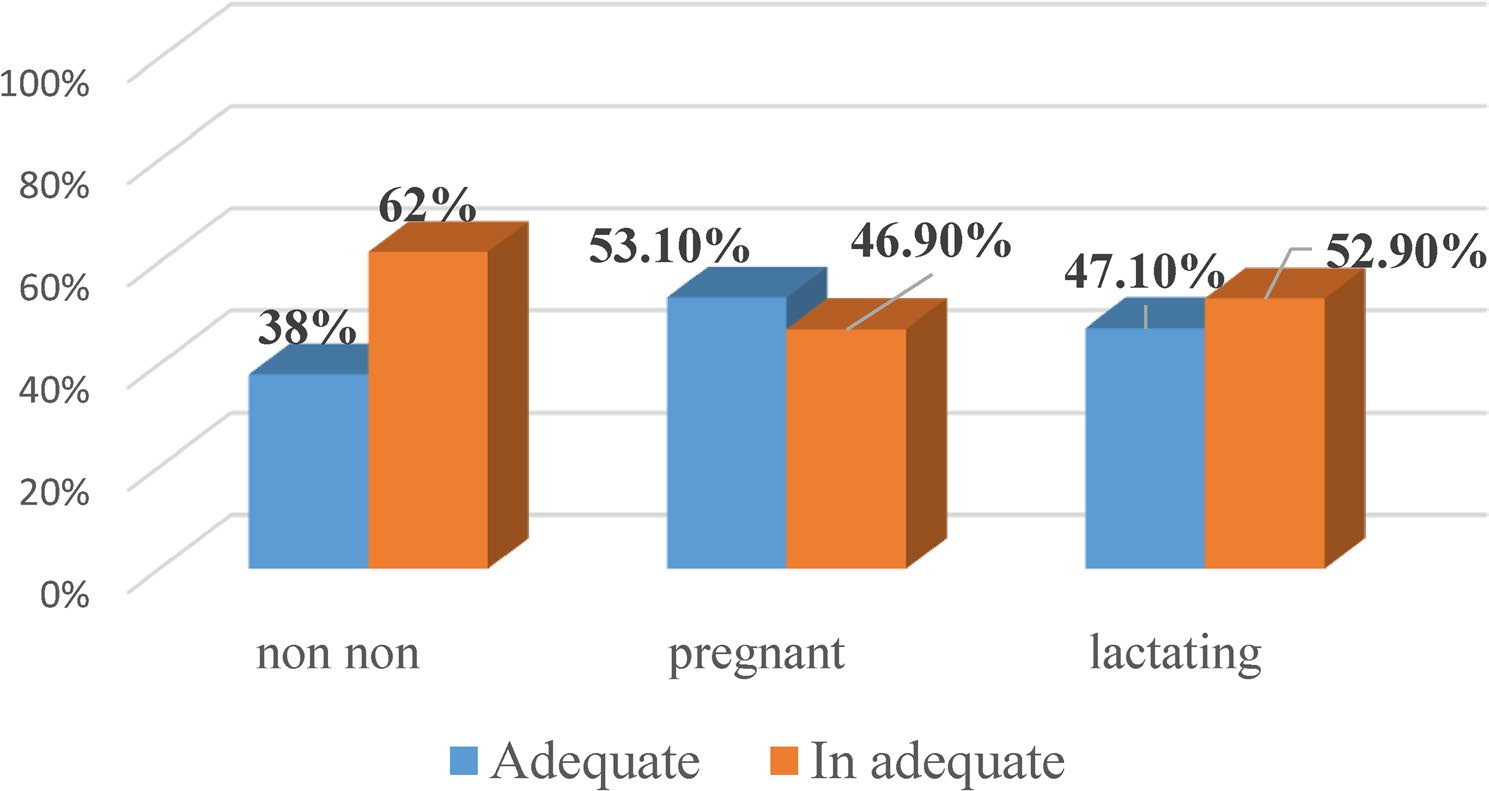
Dietary diversity among participant attending rural health care unit according to their reproductive state

Dietary diversity according to reproductive status and place of residence is depicted in Figs. 4 and 5. Among urban and rural participants around half of pregnant women (53.3%,53.1% respectively) had adequate dietary diversity (ADD). On the other hand, 60.9% of urban lactating women reported adequate dietary diversity corresponding to only 47.1% of rural ones. Moreover, the non-pregnant non-lactating women were had the lowest level of dietary diversity either at urban (48.8%) or at rural (38%) health care facilities.

These findings are supported by analytical comparisons presented in Table  2, which shows the distribution of dietary diversity adequacy across reproductive stages by residence. Urban non-pregnant non-lactating women and lactating women had significantly (*p* = 0.024, *p* = 0.005 respectively) higher dietary diversity compared with rural women within the same reproductive categories.

**Table 2:** Distribution of participant women by their reproductive state and dietary diversity at urban and rural health care facilities

**Reproductive state**	**Urban (n=213)**				**Rural (n=187)**				***p*** **- value**
	**Adequate**		**Inadequate**		**Adequate**		**Inadequate**		
	**n**	**%**	**n**	**%**	**n**	**%**	**n**	**%**	
Non-pregnant non-Lactating Women	62	48.8	65	51.2	46	38.0	75	62.0	0.024*
Pregnant women	24	53.3	21	46.7	17	53.1	15	46.9	0.825
Lactating women	25	60.9	16	39.1	16	47.1	18	52.9	0.005*

*p:<0.05 (statistically significant), Adequate: consume ≥ food groups /24hs, Inadequate: consume < 5 food groups/24hs

The consumption of the ten Minimum Dietary Diversity for Women (MDD-W) food groups is summarized in Table 3. Grains and white root vegetables were consumed by nearly all participants. Significant urban–rural differences (*p* < 0.05) were observed for several food groups, including nuts and seeds, meat and fish, vitamin A–rich fruits and vegetables, and other fruits, which were more frequently consumed by urban women. In contrast, egg, pulses, dark green leafy vegetables consumption was significantly higher (*p* < 0.05) among rural women.

**Table 3:** Ten food groups intake among participants attending urban and rural health care facilities

Ten food groups	Urban (*n* = 213)		Rural (*n* = 187)		Total (*n* = 400)		*x*^2^	*p* value
	*n*	%	*n*	%	*n*	%		
1. Grains and white root	207	97.2	186	99.5	393	98.3	3.01	0.082
2. Pulses	100	46.9	108	57.8	208	52.0	4.85	0.028*
3. Nuts and seeds	23	10.8	4	2.1	27	6.8	11.86	0.001*
4. Dairy product	150	70.4	121	64.7	271	67.8	1.62	0.20
5. Meat and fish	101	47.4	68	36.4	169	42.3	5.55	0.018*
6. Egg	67	31.5	82	43.8	149	37.3	5.03	0.025*
7. Vit A rich fruit and vegetable	33	15.5	16	8.6	49	12.3	4.48	0.034*
8. Dark green leafy vegetables	27	12.7	31	16.6	58	14.5	1.94	0.163
9. Other fruit	85	40.0	48	25.7	133	33.3	9.28	0.002*
10. Other vegetables	115	54.0	100	53.5	215	53.8	0.27	0.59

chi square test, *p:<0.05(statistically significant)

Other vegetables: Cauliflower - cucumber - green pepper - cabbage -lettuce - okra - green onions – tomatoes

Other fruit: grapes - guava - banana – apple- tangerine, cantaloupe- orange Lemon

Dairy product: Milk, cheese, yoghurt or other milk products but NOT including butter, ice cream, cream

Based on food frequency questionnaire results of Tables 4 and 5 show that food frequency patterns of grains, vegetables, and fruits were consumed most frequently, while animal-source foods and nuts/seeds were consumed less often. Urban women reported higher intake of meat, fish, and vitamin A–rich foods, whereas rural women consumed slightly more eggs. These data were used descriptively and not included in dietary diversity scoring.

**Table 4:** Food frequency consumption among participants attending urban health care center (*n* = 210)

Food frequency	Once a day or more often		3 times per week or more		1–2 times per week		Monthly		More than one month		Never	
	*n*	%	*n*	%	*n*	%	*n*	%	*n*	%	*n*	%
Grains	187	87.8	19	8.9	4	1.9	1	0.5	2	0.9	0	0.0
Pulses	32	15.0	57	26.8	83	39.0	25	11.7	12	5.6	4	1.9
Nuts and seeds	0	0.0	17	8.0	42	19.7	76	35.7	48	22.5	30	14.1
Milk and milk product	109	51.2	58	27.2	26	12.2	9	4.2	9	4.2	2	0.9
Red meat	0	0.0	11	5.2	130	61.0	40	18.8	15	7.0	17	8.0
White meat	0	0.0	58	27.2	143	67.1	6	2.8	5	2.3	1	0.5
Fish and sea food	0	0.0	3	1.4	166	77.9	34	16.0	6	2.8	4	1.9
Egg	83	39.0	70	32.9	31	14.5	20	9.4	7	3.3	2	0.9
Fruit	122	57.3	57	26.8	28	13.1	4	1.9	2	0.9	0	0.0
Vegetables	136	63.8	52	24.4	22	10.3	1	0.5	2	0.9	0	0.0
Sugary snack^*^	39	18.3	48	22.5	43	20.2	38	17.8	19	8.9	26	12.2
Salted snack**	38	17.8	49	23.6	38	18.3	40	18.3	21	9.6	27	12.4
Sweetened beverage^	128	60.1	38	17.8	23	10.8	13	6.1	3	1.4	8	3.8

*Sugary snack: sweet foods, such as candy, chocolates, cakes, sweet biscuits/cookies, sweet pastries and ice cream

**Salted snack: crisps, chips, puffs

^Sweetened beverage: Sweetened fruit juices and “juice drinks”, soft drinks, chocolate drinks, malt drinks, yoghurt drinks, sweet tea or coffee with sugar

**Table 5:** Food frequency consumption among participants attending the rural health care unit (n= 187)

Food frequency	Once a day or more often		3 times per week or more		1–2 times per week		Monthly		More than one month		Never	
	*n*	%	*n*	%	*n*	%	*n*	%	*n*	%	*n*	%
Grains	180	96.3	5	2.6	2	1.1	0	0.0	0	0.0	0	0.0
Pulses	34	18.2	60	32.1	63	33.7	21	11.2	6	3.2	3	1.6
Nuts and seeds	0	0.0	17	9.1	30	16.0	44	23.5	38	20.0	58	31.0
Milk and milk product	69	36.9	44	23.5	46	24.8	9	4.8	7	3.7	12	6.4
Red meat	0	0.0	3	1.6	73	39.0	59	31.6	21	11.2	31	16.6
White meat	1	0.5	32	17.1	139	74.3	8	4.3	3	1.6	4	2.1
Fish and sea food	0	0.0	7	3.7	145	77.5	24	12.8	8	4.3	3	1.6
Egg	79	42.2	56	29.9	34	18.2	6	3.2	7	3.7	5	2.7
Fruit	77	41.2	65	34.8	30	16.0	10	5.3	4	2.1	1	0.5
Vegetables	120	64.5	52	28.0	12	5.9	2	1.1	1	0.5	0	0.0
Sugary snack*	50	26.7	21	11.2	30	16.0	27	14.4	11	5.9	48	25.7
Salted snack**	50	26.7	27	14.4	29	15.5	23	12.3	11	5.9	47	25.1
Sweetened beverage^	118	63.1	21	11.2	23	12.3	3	1.6	4	2.1	18	9.6

*Sugary snack:sweet foods, such as candy, chocolates, cakes, sweet biscuits/cookies, sweet pastries and ice cream

**Salted snack: crisps, chips, puffs ^Sweetened beverage:Sweetened fruit juices and “juice drinks”, soft drinks, chocolate drinks, malt drinks, yoghurt drinks, sweet tea or coffee with sugar

Factors associated with inadequate dietary diversity were examined using binary logistic regression, with results presented in Table 6. Being non-pregnant, and non-lactating, (AOR: 2.370 & *P* = 0.006), having a lower educational level (AOR: 2.212 & *P* = 0.036), being unemployed (AOR:1.636 &*P* = 0.035), and reporting difficulty accessing food due to high prices AOR: 1.698 & *P* = 0.027) were independently associated with higher odds of inadequate dietary diversity.

**Table 6:** Binary logistic regression for predictors of inadequate dietary diversity among studied participants

Predicting factors of inadequate dietary diversity	B	S.E.	Wald	*P*	AOR	95% C.I.	
Univariate predictors						Lower	Upper
Residence							
Urban (Reference)							
Rural	0.289	0.234	1.518	0.218	1.335	0.843	2.113
Educational level							
High (Reference)							
Low	0.794	0.378	4.419	0.036*	2.212	1.055	4.638
Occupation							
Employed (Reference)							
Unemployed	0.492	0.233	4.470	0.035*	1.636	1.037	2.582
Income							
Enough (Reference)							
Not enough income	0.179	0.290	0.381	0.537	1.196	0.677	2.112
Reproductive stage							
Pregnant and lactating (Reference)							
Non pregnant non lactating	0.863	0.314	7.569	0.006*	2.370	1.282	4.383
Food stuff availability							
Available and easy to get it(Reference)							
Unavailability of food items due to high price	0.529	0.239	4.903	0.027*	1.698	1.063	2.712

*B *Unstandardized Coefficients, *AOR *Adjusted Odds ratio, *CI *Confidence interval, *LL *Lower limit, *UL *Upper Limit, *: Statistically significant at* p* < 5

## Discussion

Ensuring adequate nutrition for women of reproductive age (WRA) is critical not only for individual health but also for broader public health outcomes, including maternal health, child development, and intergenerational well-being. This study assessed dietary diversity among WRA attending urban and rural healthcare facilities in El Mahalla El-Kubra district, Middle Delta, Egypt, focusing on differences across reproductive stages (non-pregnant non-lactating, pregnant, lactating) and localities. To the best of our knowledge, this is among the first studies in Egypt to consider reproductive stages –specific dietary needs in examining dietary diversity.

### Overall dietary diversity

Less than half of the participants (47.5%) achieved adequate dietary diversity (≥ 5 food groups/24 hs.), reflecting generally low diet quality among WRA. This is lower than findings from the ELANS survey across Latin America, where Gómez et al., found that 57.7% of women met the MDD-W threshold [14]. This highlights the need for targeted nutrition interventions. Suboptimal dietary diversity is concerned given its implications for macro- and micronutrient adequacy and reproductive health outcomes.

### Urban–rural differences

Urban women consistently exhibited higher dietary diversity than rural ones, a finding consistent with studies from Northern Uganda (Oyet et al., 2023) [15] and Mauritania (Janmohamed et al., 2024) [16]. Urban participants consumed more animal-source foods, nuts, and vitamin A–rich fruits and vegetables, whereas rural women had higher intake of eggs and pulses. These differences likely reflect disparities in food availability, affordability, market access, and nutrition knowledge. Local food environments and cultural dietary patterns may further shape consumption, particularly in rural areas where limited access and higher food prices were reported as barriers.

### Reproductive-stage differences

Stratification by reproductive stage revealed important disparities. Lactating women had the highest dietary diversity (54.7%), particularly in urban areas (60.9%), whereas non-pregnant, non-lactating women reported the lowest adequacy (43.5% overall; 38.8% in rural areas, 48.8% in urban areas, *p* = 0.024). These findings underscore the vulnerability of non-pregnant, non-lactating women, a group less frequently addressed in nutrition research, and highlight the importance of extending interventions beyond pregnancy and lactation.

Pregnancy is a significant and nutritionally demanding period of a woman’s life because the physiological changes that ensure foetal growth and development lead to higher nutritional demands during pregnancy [17]. At the current study, only half of cases across urban and rural settings had adequate diversed diet, indicating ongoing gaps in meeting nutritional needs despite health facility attendance. This may reflect gaps in the quality or frequency of nutrition counseling during antenatal visits, cultural food taboos that restrict certain foods during pregnancy, and economic constraints limiting access to diverse foods. Strengthening facility-based nutrition education and addressing socio-cultural barriers are essential to improve dietary diversity among pregnant women.

The low level of dietary diversity among pregnant women at the present study at both urban and rural setting and was consistent with findings of Yeneabat et al., Mesfin et al., and Wondmeneh from Ethiopia [18–20]. In contrast, Uwase et al. from Rwanda revealed that rural pregnant women were more diverse (47.0%) than urban (41.1%), most likely due to home gardening [21]. At the present study, rural women identified food prices and access as a barrier.

Food diversity and adequacy in the lactation period are fundamental for maternal and child health. Lactating mothers are vulnerable to malnutrition because of increased physiological demand, monotonous diet, lactogenesis process, and increased nutrient requirements. More than half (54.7%) of lactating participants at the current study attained adequate dietary diversity and urban women showed more DD (60.9%) than rural ones (52.9%). This was much higher than the reported DD (38.8%) among lactating women in Southwest Ethiopia [22]. On the other hand, in India, Shumayla et al. found that 77.1% of lactating women experienced adequate DD and urban settings reported greater diversity than rural women [23]. Together, these findings highlight how context, stage of reproduction, and access to resources shape women’s diets differently across settings.

### Food group patterns

Analysis of specific food groups provided additional insights. Grains and white roots dominated diets across both settings, consistent with findings of Darroudi et al., from Iran [24]. Urban women consumed more dairy, meat, nuts, and vitamin A-rich fruits and vegetables, whereas rural women consumed more pulses, eggs, and leafy greens which are more available in rural culture, and this was in line with studies from Cameroon and Kenya [25] and Iran (Darroudi et al., [24]. Also, this was consistent with evidence from African countries like Benue, Cameroon and Senegal [25] and Uganda [26], besides Thailand in Asia [27].

Low consumption of nuts and seeds in rural areas contrasts with findings of Weerasekara et al., from Sri Lanka [28], illustrating contextual variability. High cereal consumption combined with low intake of animal-source foods may compromise iron and zinc bioavailability.

### Socioeconomic determinants

Education, employment, and food availability were significant predictors of dietary diversity. Women with university education were substantially more likely to achieve adequacy, while those with lower attainment were nearly twice as likely to report inadequate diets, consistent with studies from Bangladesh [29]) and China [30]. Government-employed women exhibited the highest adequacy, whereas unemployed women were 1.6 times more likely to have insufficient diversity, aligning with evidence reported by Bikila et al., from Ethiopia [5] and Mekonen from three Sub-Saharan African and South Asian countries [31]. Higher income and better food access were also associated with greater dietary diversity, corroborating findings from South Africa [32]), Ethiopia [33], and other LMICs [34].

#### Strengths and limitations

##### Strengths

Novelty and relevance: this study is one of the first studies in Egypt that describe the dietary diversity among reproductive-aged women at different reproductive stages(non-pregnant/non-lactating, pregnant, lactating) in both urban and rural settings. So, it addresses a significant public health need by concentrating on women who are not pregnant or lactating, which are frequently neglected in nutritional studies. The use of two validated tools; DD score and FFQ complement each other to give relatively good idea about the individual’s habitual diet. Also, the study include a relatively large size sample selected randomly by systematic random sampling which reduce selection bias and empower presentiveness. Moreover,the study follows the STROBE criteria of transparency, replication, and scientific rigor.

### Limitations

Cross-sectional design: limits the ability to establish causal relationships between dietary diversity and different variables. Recalling and reporting bias: self-reported dietary data may be affected by memory and social desirability. Single 24-h recall may not reflect usual intake; FFQ lacked portion size assessment. Unmeasured confounding factors such as BMI, physical activity, or health status were not assessed. Nonresponse and missing data: Not reported, which could affect representativeness.

#### Conclusion and recommendations

Over half of WRA had inadequate dietary diversity, with non-pregnant, non-lactating women and rural residents most affected. Key predictors of inadequate diversity included low education, unemployment, reproductive stage, and limited food availability. Addressing these gaps requires context-specific strategies:

- Strengthening rural nutrition outreach and women-centered nutrition clinics.
- Promoting local production of nutrient-dense foods.
- Stage-specific dietary programs including health education for non-pregnant, non-lactating women.
- Enhanced antenatal and postnatal nutrition counseling.
- Policies to improve employment, income, and market access.

These interventions are essential to improve maternal nutrition, support healthy pregnancy outcomes, and enhance intergenerational health. 

## Supplementary Information

Supplementary Material 1.

Supplementary Material 2.

Supplementary Material 3.

## Data Availability

Data is available on reasonable request from the corresponding author.

## Abbreviations

ADD: Adequate dietary diversity
DD: Dietary Diversity
FFQ: Food Frequency Questionnaire
LMICs: Low- and Middle-Income Countries
MDD-W: Minimum Dietary Diversity for Women
SPSS: Statistical Package for Social Sciences
WRA: Women of Reproductive Age

## References

[1] Abebe A. Prevalence of dietary diversity practice and associated factors among women of reproductive age in Asaita districts, Ethiopia. Mathews J Nutr Diet. 2024;7(3):1–14.

[2] Silvestris E, Lovero D, Palmirotta R. Nutrition and female fertility: an interdependent correlation. Front Endocrinol (Lausanne). 2019;10:346. 10.3389/fendo.2019.00346. 31231310 10.3389/fendo.2019.00346 PMC6568019

[3] Islam MH, Nayan MM, Jubayer A, Amin MR. A review of the dietary diversity and micronutrient adequacy among the women of reproductive age in low- and middle-income countries. Food Sci Nutr. 2024;12(3):1367–79. 10.1002/fsn3.1367. 38455218 10.1002/fsn3.3855 PMC10916566

[4] Nkoko N, Cronje N, Swanepoel JW. Determinants of dietary diversity for women of reproductive age (WRA) and under-five children from small-holder farming households in Lesotho. Cogent Food Agric. 2023;9(1):2231688. 10.1080/23311932.2023.2231688.

[5] Bikila H, Ariti BT, Fite MB, Sanbata JH. Prevalence and factors associated with adequate dietary diversity among pregnant women in Nekemte town, Western Ethiopia, 2021. Front Nutr. 2023;10:1248974. 10.3389/fnut.2023.1248974. 38162525 10.3389/fnut.2023.1248974 PMC10756138

[6] Saleh S, Elsayed H, El Gezery H, Mostafa A. Micronutrient intake profile of Egyptian women in reproductive ages. Bull Natl Nutr Inst Arab Repub Egypt. 2022;60(2):212–41.

[7] Ruel MT. Operationalizing dietary diversity: a review of measurement issues and research priorities. J Nutr. 2003;133(11 Suppl 2):S3911–26. 10.1093/jn/133.11.3911S 14672290

[8] Difference between balanced diet and adequate diet; their significance. Icliniq. 2022. Available from: https://wellness.icliniq.com/articles/diet-and-nutrition/difference-between-balanced-diet-and-adequate-diet. Accessed 17 Dec 2025.

[9] Balanced diet: deficiency and diseases. Embibe. 2023. Available from: https://www.embibe.com/exams/balanced-diet/. Accessed 17 Dec 2025.

[10] Ghaferi AA, Schwartz TA, Pawlik TM. STROBE reporting guidelines for observational studies. JAMA Surg. 2021;156(6):577–8. 10.1001/jamasurg.2021.1294. 33825815 10.1001/jamasurg.2021.0528

[11] FAO. FHI. Minimum Dietary Diversity for Women: A Guide for Measurement. Rome: FAO; 2016. p. 360.

[12] FAO. Minimum dietary diversity for women: an updated guide to measurement – from collection to action. Rome: FAO; 2021.

[13] Rothenberg E, Strandhagen E, Samuelsson J, et al. Relative validity of a short 15-item food frequency questionnaire measuring dietary quality, by the diet history method. Nutrients. 2021;13(11):3754. 10.3390/nu13113754. 34836011 10.3390/nu13113754 PMC8622557

[14] Gómez G, Nogueira Previdelli Á, Fisberg RM, Kovalskys I, Fisberg M, Herrera-Cuenca M, et al. Dietary diversity and micronutrient adequacy in women of childbearing age: results from ELANS study. Nutrients. 2020;12(7):1994. 10.3390/nu12071994. 32635544 10.3390/nu12071994 PMC7400493

[15] Kolliesuah NP, Olum S, Ongeng D. Status of household dietary diversity and associated factors among rural and urban households of Northern Uganda. BMC Nutr. 2023;9(1):83. 10.1186/s40795-023-00739-4. 37430346 10.1186/s40795-023-00739-4 PMC10332000

[16] Issa MY, Diagana Y, Khalid ELK, et al. Dietary diversity and its determinants among women of reproductive age residing in the urban area of Nouakchott, Mauritania. BMC Public Health. 2024;24(1):916. 10.1186/s12889-024-14045-2. 38549049 10.1186/s12889-024-18211-8 PMC10979579

[17] WHO. WHO recommendations on antenatal care for a positive pregnancy experience. Geneva: World Health Organization. 2016. Available from: https://www.who.int/publications/i/item/9789241549912 28079998

[18] Yeneabat T, Adugna H, Asmamaw T, et al. Maternal dietary diversity and micronutrient adequacy during pregnancy and related factors in East Gojjam Zone, Northwest Ethiopia. BMC Pregnancy Childbirth. 2019;19:1–9. 10.1186/s12884-019-2317-4. 31092223 10.1186/s12884-019-2299-2 PMC6521398

[19] Mesfin S, Abebe D, Jiru HD, Sori SA. Only two in five pregnant women have adequate dietary diversity during antenatal care at Hiwot Fana Specialized University Hospital in Eastern Ethiopia. J Nutr Sci. 2024;13:e17. 10.1017/jns.2024.7. 38572370 10.1017/jns.2024.7 PMC10988145

[20] Wondmeneh TG. Dietary diversity practice and its influencing factors among pregnant women in Afar region of Ethiopia: mixed method study. BMC Pregnancy Childbirth. 2022;22:291. 10.1186/s12884-022-04613-0. 35387620 10.1186/s12884-022-04641-y PMC8988420

[21] Uwase A, Nsereko E, Pillay N, Levin J. Dietary diversity and associated factors among pregnant women in the Southern Province of Rwanda: A facility-based cross-sectional study. PLoS ONE. 2024;19(2):e0297112. 10.1371/journal.pone.0297112. 38394158 10.1371/journal.pone.0297112 PMC10889653

[22] Teferi T, Endalk G, Ayenew GM, et al. Inadequate dietary diversity practices and associated factors among postpartum mothers in Gambella town, Southwest Ethiopia. Sci Rep. 2023;13:7252. 10.1038/s41598-023-29962-6. 37142603 10.1038/s41598-023-29962-6 PMC10160103

[23] Shumayla S, Irfan EM, Kathuria N, et al. Minimum dietary diversity and associated factors among lactating mothers in Haryana, India: a community based cross-sectional study. BMC Pediatr. 2022;22:525. 10.1186/s12887-022-03588-5. 36057585 10.1186/s12887-022-03588-5 PMC9440519

[24] Darroudi S, Soflaei SS, Kamrani F, et al. Urban and rural residence: their influence on food group consumption in Iran. BMC Public Health. 2025;25:169. 10.1186/s12889-025-13258-9. 39815251 10.1186/s12889-024-21211-3 PMC11736970

[25] Janmohamed A, Baker MM, Doledec D, et al. Dietary quality and associated factors among women of reproductive age in six Sub-Saharan African countries. Nutrients. 2024;16(8):1115. 10.3390/nu16081115. 38674806 10.3390/nu16081115 PMC11054593

[26] Oyet SM, Kaahwa RM, Muggaga C, et al. Household dietary diversity and associated factors in rural and peri-urban areas of Mbale District, Eastern Uganda. BMC Public Health. 2025;25:303. 10.1186/s12889-025-13456-2. 39856627 10.1186/s12889-025-21476-2 PMC11761235

[27] Puwanant M, Boonrusmee S, Jaruratanasirikul S, et al. Dietary diversity and micronutrient adequacy among women of reproductive age: a cross-sectional study in Southern Thailand. BMC Nutr. 2022;8:127. 10.1186/s40795-022-00633-1. 36348450 10.1186/s40795-022-00619-3 PMC9641308

[28] Weerasekara PC, Withanachchi CR, Ginigaddara GAS, et al. Understanding dietary diversity, dietary practices and changes in food patterns in marginalized societies in Sri Lanka. Foods. 2020;9(11):1659. 10.3390/foods9111659. 33202762 10.3390/foods9111659 PMC7696452

[29] Islam MH, Jubayer A, Nowar A, et al. Dietary diversity and micronutrients adequacy among the women of reproductive age at St. Martin’s island in Bangladesh. BMC Nutr. 2023;9:52. 10.1186/s40795-023-00756-0. 36945035 10.1186/s40795-023-00715-y PMC10029180

[30] Deng C, Vicerra PMM. Household structure and dietary diversity among older adults in rural and urban China: a cross-sectional study. BMC Public Health. 2024;24:3004. 10.1186/s12889-024-14678-3. 39478515 10.1186/s12889-024-20434-8 PMC11523886

[31] Mekonen EG. Minimum dietary diversity and its determinants among women of childbearing age in three Sub-Saharan African and South Asian countries: evidence from the most recent nationally representative surveys (2022). Women’s Health Rep. 2024;5:954–64. 10.1089/whr.2024.0052.

[32] Chakona G, Shackleton CM. Minimum dietary diversity scores for women indicate micronutrient adequacy and food insecurity status in South African towns. Nutrients. 2017;9:812. 10.3390/nu9080812. 28788057 10.3390/nu9080812 PMC5579606

[33] Merga G, Mideksa S, Dida N, Kennedy G. Dietary diversity and associated factors among women of reproductive age in Jeldu District, West Shoa Zone, Oromia Ethiopia. PLoS ONE. 2022;17:e0264231. 10.1371/journal.pone.0264231. 36534691 10.1371/journal.pone.0279223 PMC9762567

[34] Choudhury S, Bi AZ, Medina-Lara A, et al. The rural food environment and its association with diet, nutrition status, and health outcomes in low-income and middle-income countries (LMICs): a systematic review. BMC Public Health. 2025;25:1–18. 10.1186/s12889-025-13789-4. 40082817 10.1186/s12889-025-22098-4 PMC11907969

