# Supplementary material for: Dietary diversity among reproductive aged women attending urban and rural healthcare facilities, Middle Delta, Egypt
Source: BMC Public Health. 2026 Apr 2;26:1174. doi: 10.1186/s12889-026-26977-2 (PMC13064081; doi:10.1186/s12889-026-26977-2)
Supplement: Supplementary file 1 — Supplementary Material 1. [file 12889_2026_26977_MOESM1_ESM.pdf]

Dietary diversity among reproductive aged women: Comparative study between an urban and rural attendants of health care facilities, Middle Delta, Egypt

(1) Socio-demographic characteristics:

- **Age**:
- **Residence:**

-Urban - Rural

- **Marital status**:

-Single -Married -Divorce -Widow

- **Educational level:**

-Illiterate -Read and write - Basic education Secondary/ Diploma -University level

- **Occupation**:

-House wife - Manual worker - Governmental employee

- Private employee -Students

Availability of food staff:

Available and easy to get it Difficult due to high prices

Current reproductive status:

Non pregnant non lactating pregnant lactating

Code: Age: Height: Weight: Serial number:

24 H dietary recall

Meals	Consumed foods		Quantity (household measure)	Quantity (grams)	code	Notes
	Ingredients	items				

Modified Dietary diversity questionnaire:

	Food categories	Description/examples to be adapted	Consumed Yes = 1 No= 0
A	Foods made from grains	bread, rice, pasta/noodles or other foods made from grains	__ yes (1) ___ no (0)
B	White root/ tuber	Potatoes, Taro	__ yes (1) ___ no (0)
C	Pulses (beans, peas and lentils)	Mature beans or peas (fresh or dried seed), lentils or bean/pea products	__ yes (1) ___ no (0)
D	Nuts and seeds	peanut, sesame or nut/seed "butters"	__ yes (1) ___ no (0)
E	Milk and milk products	Milk, cheese, yoghurt or other milk products but NOT including butter, ice cream, cream	__ yes (1) ___ no (0)
F	Organ meat<br>Processed meat<br>Red meat<br>White meat<br>Fish and seafood	Liver, kidney, heart or other organ meats<br>Luncheon - sausage<br>Beef, goat, duck and pigeon<br>rabbit, chicken<br>Fresh, dried fish, salted fish, canned fish and seafood (shrimp and crab)	__ yes (1) ___ no (0)
I	Eggs	Eggs from poultry or any other bird	__ yes (1) ___ no (0)
J	Dark green leafy vegetables	Spinach, dark green leafy lettuce , mukhiya,…..	__ yes (1) ___ no (0)

K	Vitamin A-rich vegetables<br>Vitamin A-rich fruits	Red pepper, carrots, sweet potatoes<br>Apricot, mango, …..	__ yes (1) ___ no (0)
M	Other vegetables	Cauliflower - cucumber - green pepper - cabbage -lettuce - okra - green onions – tomatoes… . .	__ yes (1) ___ no (0)
N	Other fruits	Grapes - guava - banana – apple-tangerine, cantaloupe- orange -Lemon ,….-	yes (1) ___ no (0)

Foods frequency Questionnaire:

Food groups	Once a day or more often	3 times per week or more	1-2 times per week	Monthly	never
Red meat					
White meat					
Fish and sea food					
Egg					
Milk and milk product					
Nuts and seeds					
Fruit					
Vegetables					
Grains					
Pulses					
sweetened beverage					
snacks					
