# Supplementary material for: Dietary diversity among reproductive aged women attending urban and rural healthcare facilities, Middle Delta, Egypt
Source: BMC Public Health. 2026 Apr 2;26:1174. doi: 10.1186/s12889-026-26977-2 (PMC13064081; doi:10.1186/s12889-026-26977-2)
Supplement: Supplementary file 2 — Supplementary Material 2. [file 12889_2026_26977_MOESM2_ESM.pdf]

التنوع الغذائي بين النساء في سن الإنجاب: دراسة مقارنة بين مرافق الرعاية الصحية في المناطق الحضرية والريفية، وسط الدلتا، مصر

(1)الخصائص الاجتماعية والديموغرافية:

- **العمر:**
- **مكان الإقامة:**
  - حضري
  - ريفي
- **الحالة الاجتماعية:**
  - أعزب
  - متزوج
  - مطلق
  - أرمل
- **المستوى التعليمي:**
  - أمي
  - يقرأ ويكتب
  - تعليم أساسي
  - ثانوي / دبلوم
  - مستوى جامعي
- **المهنة:**
  - ربة منزل
  - عامل يدوي
  - موظف حكومي
  - موظف قطاع خاص
  - طالب
- **توفر المواد الغذائية:**
  - متوفرة ويسهل الحصول عليها
  - صعبة بسبب ارتفاع الأسعار
- **الحالة الإنجابية الحالية:**
  - غير حامل وغير مرضع
  - حامل
  - مرضع

الاسم:　　　　السن:　　　　رقم المسلسل:　　　　الوزن:　　　　الطول:

الحاله الانجابيه

استرجاع 24 ساعه

الوجبات	الاطعمه المتناوله		الكود	الكميه (مكيال)	الكميه بالجرام	ملاحظات
	الصنف	المكونات				

فئة الطعام	الوصف / أمثلة للتوضيح	مستهلك نعم = 1 لا = 0
1	أطعمة مصنوعة من الحبوب: خبز، أرز، مكرونة/نودلز أو غيرها من الأطعمة المصنوعة من الحبوب	__نعم (1) __ لا(0)
	جذور ودرنات بيضاء: بطاطس، قلقاس	__نعم (1) __ لا(0)
2	البقوليات (الفول، البازلاء والعدس): الفول أو البازلاء الناضجة (طازجة أو مجففة)، العدس أو منتجات الفول/البازلاء	__نعم (1) __ لا(0)
3	المكسرات والبذور: فول سوداني، سمسم أو زبدة المكسرات/البذور	__نعم (1) __ لا(0)
4	الحليب ومنتجات الألبان: حليب، جبن، زبادي أو منتجات ألبان أخرى (باستثناء الزبدة، الآيس كريم، القشطة)	__نعم (1) __ لا(0)
5	اللحوم والأعضاء الداخلية واللحوم المصنعة: كبد، كلى، قلب أو أعضاء أخرى / لانشون – سجق / لحم بقري، ماعز، بط، حمام / أرنب، دجاج / أسماك طازجة، مجففة، مملحة، معلبة، مأكولات بحرية (جمبري، كابوريا)	__نعم (1) __ لا(0)
6	البيض: بيض الدواجن أو أي طيور أخرى	__نعم (1) __ لا(0)
7	الخضروات الورقية الخضراء الداكنة: سبانخ ، ملوخية أو أي خضروات أخرى مثلهم	__نعم (1) __ لا(0)
8	الخضروات الغنية بفيتامين أ: فلفل أحمر، جزر، بطاطا حلوة / الفواكه الغنية بفيتامين أ: مشمش، مانجو	__نعم (1) __ لا(0)
9	خضروات أخرى: قرنبيط، خيار، فلفل أخضر، كرنب، خس، بامية، بصل أخضر، طماطم	__نعم (1) __ لا(0)
10	فواكه أخرى: عنب، جوافة، موز، تفاح، يوسفي، شمام، برتقال، ليمون	__نعم (1) __ لا(0)

تكرار استهلاك الطعام:

مجموعات الطعام	مرة يومياً أو أكثر	3مرات أسبوعياً أو أكثر	1-2مرات أسبوعياً	شهرياً	أبداً
اللحوم الحمراء					
اللحوم البيضاء					
الأسماك والمأكولات البحرية					
البيض					
الحليب ومنتجات الألبان					
المكسرات والبذور					
الفواكه					
الخضروات					
الحبوب					
البقوليات					
المشروبات المحلاة					
الوجبات الخفيفة					
