# Supplementary material for: Dietary diversity among reproductive aged women attending urban and rural healthcare facilities, Middle Delta, Egypt
Source: BMC Public Health. 2026 Apr 2;26:1174. doi: 10.1186/s12889-026-26977-2 (PMC13064081; doi:10.1186/s12889-026-26977-2)
Supplement: Supplementary file 3 — Supplementary Material 3. [file 12889_2026_26977_MOESM3_ESM.docx]

| **STROBE Item** | **Location in paper** | **Page(s)** |
| --- | --- | --- |
| Title & Abstract | Title, Abstract (study design mentioned) | 1,2 |
| Background/Rationale | Introduction – background and rationale | 3,4 |
| Objectives | objectives | 4 |
| Study Design | Methods – Study design (cross-sectional) | 4 |
| Setting | Methods – Study setting and duration (urban/rural) | 5 |
| Participants | Methods – Eligibility, sampling strategy | 5 |
| Variables | Methods – Sociodemographic, MDD-W | 6 |
| Data Sources/Measurement | Methods – 24h recall, FFQ | 6-7 |
| Bias | Methods – Pilot study, systematic sampling; Limitations | 6,8,20 |
| Sample Size | Methods – Sample size calculation (N=400) | 6 |
| Statistical Methods | Methods – Chi-square, Binary regression | 9 |
| Participants (Flow) | Results – Participants description (urban/rural, stage) | 9 |
| Descriptive Data | Results – Table 1 (sociodemographic) | 10 |
| Outcome Data | Results – Tables 2–6, Figures 1–5 (dietary diversity) | 9–16 |
| Main Results | Results – regression, ORs; Discussion | 13–14 |
| Other Analyses | Results – subgroup analysis (stage, location) | 14 |
| Key Results | Discussion – opening section | 16 |
| Limitations | Discussion – Limitations | 19 |
| Interpretation | Discussion – comparison with literature | 14-17 |
| Generalizability | Strengths | 19 |
| Funding & Ethics | Methods – Ethical considerations | 8-20 |

**Dietary diversity among reproductive aged women attending urban and rural**

**healthcare facilities, Middle Delta, Egypt**

**STROBE Checklist Validation**

| **STROBE Item** | **Fulfilled?** | **Details** |
| --- | --- | --- |
| **Title & Abstract** | ✔️ | Clearly states study design and summarizes background, methods, results, and conclusions. |
| **Background & Objectives** | ✔️ | Provides strong rationale, literature review, and clearly defined objectives. |
| **Study Design** | ✔️ | Cross-sectional design explicitly described. |
| **Setting** | ✔️ | Urban and rural health facilities in El-Mahalla El-Kubra district. |
| **Participants** | ✔️ | Inclusion/exclusion criteria defined; systematic random sampling used. |
| **Variables** | ✔️ | Sociodemographic, reproductive status, dietary diversity (MDD-W), nutrient adequacy (DRIs), BMI, clinical signs. |
| **Data Sources & Measurement** | ✔️ | Validated questionnaire, 24-hour recall, FFQ, anthropometric and clinical assessments. |
| **Bias** | ✔️ | Pilot study conducted; systematic sampling reduces selection bias. |
| **Study Size** | ✔️ | Sample size calculated statistically (N=400). |
| **Quantitative Variables** | ✔️ | Detailed measurement and analysis using SPSS. |
| **Statistical Methods** | ✔️ | Chi-square, t-tests, logistic regression; significance level set at 0.05. |
| **Participants Flow** | ✔️ | Described with breakdown by urban/rural and reproductive status. |
| **Descriptive Data** | ✔️ | Tables and figures show sociodemographic and dietary data. |
| **Outcome Data** | ✔️ | Prevalence of inadequate dietary diversity, nutrient intake, BMI, clinical signs. |
| **Main Results** | ✔️ | Regression analysis identifies key predictors (e.g., unemployment, low education). |
| **Other Analyses** | ✔️ | Subgroup comparisons by reproductive status and location. |
| **Key Results Summary** | ✔️ | Urban women had better dietary diversity and nutrient intake. |
| **Limitations** | ✔️ | Recall bias, lack of biochemical validation, seasonal variation not accounted for. |
| **Interpretation** | ✔️ | Compared with literature; implications for policy and practice discussed. |
| **Generalizability** | ✔️ | Applicable to similar urban/rural populations in Egypt. |
| **Ethical Approval** | ✔️ | Approved by Tanta Faculty of Medicine Ethics Committee. |
| **Funding** |  |  |
| **Recommendations** | ✔️ | Nutrition clinics, outreach, education, food access improvements. |
